# Expanding access to medications for opioid use disorder through locally-initiated implementation

**DOI:** 10.1186/s13722-022-00312-7

**Published:** 2022-06-20

**Authors:** Jessica J. Wyse, Katherine Mackey, Travis I. Lovejoy, Devan Kansagara, Anais Tuepker, Adam J. Gordon, P. Todd Korthuis, Anders Herreid-O’Neill, Beth Williams, Benjamin J. Morasco

**Affiliations:** 1 Center to Improve Veteran Involvement in Care, VA Portland Health Care System, 3710 SW U.S. Veterans Hospital Rd., Portland, OR 97239 USA; 2 School of Public Health, Oregon Health & Science University-Portland State University, 1810 SW 5th Avenue, Portland, OR 97201 USA; 3 Department of Psychiatry, Oregon Health & Science University, 3181 SW Sam Jackson Park Rd, Portland, OR 97239 USA; 4 Department of General Internal Medicine & Geriatrics, Oregon Health & Science University, 3181 SW Sam Jackson Park Road, Portland, OR 97239 USA; 5 Informatics, Decision-Enhancement, and Analytic Sciences (IDEAS) Center, VA Salt Lake City Health Care System, 500 Foothill Drive, Salt Lake City, UT 84148 USA; 6 Division of Epidemiology, Department of Internal Medicine, University of Utah School of Medicine, 295 Chipeta Way, Salt Lake City, UT 84132 USA; 7 Section of Addiction Medicine, Oregon Health & Science University, 3181 SW Sam Jackson Park Road, Portland, OR 97239 USA

**Keywords:** Implementation, Opioid use disorder, Medication treatment, Veterans

## Abstract

**Background:**

Despite demonstrated efficacy, medication treatment for opioid use disorder (MOUD) remain inaccessible to many patients, with barriers identified at the individual, clinic and system level. A wide array of implementation strategies have guided efforts to expand access to MOUD, with most centered around externally-facilitated approaches to practice change. While effective, such approaches may be inaccessible to those clinics and systems that lack the resources necessary to partner with an external team, suggesting a need to identify and describe change-processes that are internally developed and promoted.

**Methods:**

Guided by the Consolidated Framework for Implementation Research (CFIR), we utilized qualitative interviews and ethnographic observation to investigate the planning, design and implementation of a locally-initiated process to expand access to MOUD within one health care system. All study documents were coded by a primary coder and secondary reviewer using a codebook designed for use with the CFIR. To analyze data, we reviewed text tagged by key codes, compared these textual excerpts both across and within documents, and organized findings into themes. Processes identified were mapped to established implementation science constructs and strategies.

**Results:**

Interviews with clinicians and administrators (n = 9) and ethnographic observation of planning meetings (n = 3) revealed how a self-appointed local team developed, established broad support for, and successfully implemented a Primary Care-based Buprenorphine Clinic and E-Consult Service to expand access to MOUD to patients across the health care system. First, national and local policy changes—including altered clinical practice guidelines, performance pay incentives regarding opioid prescribing, and a directive from VA Central Office increased individual staff and administrators’ perception of the need for change and willingness to invest time and resources. Then, a self-appointed interdisciplinary team utilized cross-clinic meetings and information gathering to identify appropriate, and widely supported, models of care delivery and care consultation. Finally, the team increased staff investment in these change efforts by bringing them into the planning process and encouraging collaborative problem solving.

**Conclusions:**

This study reveals how a local team developed and built widespread support for new processes of care that were tailored to local needs and well-positioned for sustainability over time.

**Supplementary Information:**

The online version contains supplementary material available at 10.1186/s13722-022-00312-7.

## Background

Although the U.S. Federal government has spent billions of dollars on efforts to address the ongoing opioid crisis [1], the number of Americans dying from opioid-related overdose deaths continues to rise, with more than 70,000 such deaths recorded in 2020 [2]. In response, health care systems have increasingly sought to expand patient access to medications for opioid use disorder (MOUD), which are considered the gold-standard treatment for OUD [3, 4]. Patients receiving MOUD, including buprenorphine and methadone, show greater abstinence, improved retention in treatment and reduced mortality relative to those who do not receive MOUD [5, 6]. Further, both clinical trials and observational research have shown MOUD to be more effective than behavioral treatments alone in preventing serious opioid-related acute care events, relapse and overdose [5, 7–9]. In spite of the recognized benefits, MOUD remains inaccessible to many [6, 10, 11]. Within the Veterans Health Administration, the largest provider of substance use disorder (SUD) treatment within the United States, just 45% of those diagnosed with OUD received MOUD in 2021, and access to medication remains highly variable across facilities [12].

Barriers to increasing patient access to MOUD have been identified at the individual, clinic and system levels [13, 14]. Mackey et al. [20] identified patient and provider stigma regarding medication treatment for OUD and substance use disorder treatment experiences as two key individual-level barriers to MOUD prescribing (in the case of providers) and utilization (in the case of patients). At the clinic and system level, primary barriers to increased MOUD prescribing include logistical issues such as lack of time, increased costs, regulatory barriers and issues relating to insurance, such as low rates of reimbursement and a need for prior authorization. Others have identified abstinence-only treatment philosophy, lack of support from leadership, inadequate mental health and psychosocial support and lack of education about MOUD as important barriers to prescribing at the provider and clinic level [15–21].

A wide array of implementation strategies have guided efforts to overcome these barriers to MOUD access, with most centered around externally-facilitated approaches to practice change  [3, 4, 10, 15, 22–24]. Implementation strategies targeting barriers identified at the individual level (e.g., lack of medication or addiction-treatment knowledge, stigmatized beliefs, training requirements) and clinic-level (e.g., staffing, space, referral process) have included implementation facilitation, consult services and coaching, academic detailing, outreach visits, designating a champion and team based approaches to MOUD provision [3, 15, 22, 23, 25]. Strategies targeting barriers at the level of the health system (e.g., cultural change, telehealth capability, education) have included webinars, train-the-trainer initiatives, learning collaboratives, sponsored conferences and the dissemination of new clinical practice guidelines, webinars and toolkits [4, 23, 26].

While such strategies are often effective, there may be limitations to change efforts that are externally initiated. For instance, not all clinics and systems have the resources to partner with an external team to facilitate a change-process. Change may also not be sustainable once the external team’s support is withdrawn—although facilitation processes that partner with embedded, internal facilitators help to address this concern [27]. These possibilities suggest a need to identify and describe change-processes that are internally developed and promoted as a first step to understanding how such efforts may differ from those that are initiated via external partnerships.

We describe the processes and strategies through which a self-appointed team embedded within one health care system identified an unmet clinical need, developed new care processes to meet that need and increased support for these efforts over time. To provide analytic leverage for future comparative research, we mapped the processes we identified to Consolidated Framework for Implementation Research (CFIR) constructs [28, 29] and Expert Recommendations for Implementing Change (ERIC) implementation strategies [30]—a comprehensive list of strategies compiled and defined by implementation and clinical experts to establish consistent terms and definitions for implementation studies—and discuss the implication of our findings for implementation science more broadly.

## Methods

This study was conducted at the VA Portland Health Care System, a tertiary care hospital that provides ongoing care to nearly 100,000 patients. This system includes 12 primary care clinic locations, but the described interventions specifically took place within the VA Portland Resident & Faculty Primary Care Clinic, an academic primary care clinic that serves approximately 6000 patients and is staffed by 15 faculty members and approximately 50 Internal Medicine residents. This clinic is located on the same campus as the hospital and many specialty care clinics, including substance use disorder and pain management clinics and an opioid treatment program. Qualitative data collection took place between July of 2019 and September of 2020 and included ethnographic observations of planning meetings and semi-structured interviews with clinicians and leadership engaged in the planning and implementation of efforts to expand access to MOUD. The study was approved by the joint Institutional Review Board at the VA Portland Health Care System and Oregon Health and Science University.

The CFIR informed key aspects of study design and analysis. CFIR is an inclusive typology of five overarching domains and affiliated constructs that have been found to influence implementation processes and outcomes. CFIR domains and examples of underlying constructs include: inner setting (e.g., networks and communication), outer setting (e.g., external policies and incentives), intervention characteristics (e.g., adaptability), process (e.g., engaging) and characteristics of individuals (e.g. individual stage of change) [28, 29].

Potential interview subjects were suggested by a study author integral to the planning efforts and included all clinicians and administrators involved in the development of the new care processes. Subjects included clinical pharmacists, physicians, and RNs. The study PI reached out to clinicians and administrators either in person or via email to participate in a single individual qualitative interview. All of those contacted agreed to participate. The interviewer (and study PI) was a PhD-level social scientist embedded in the clinical setting in which the implementation took place. As such, the interviewer was known to many of the interview respondents. Interviews were conducted in a private office within the Medical Center or over the phone (following altered procedures after the onset of COVID-19). All interviewees provided informed consent to participate. Interviews lasted between 30 and 90 min. The interview protocol was informed by prior literature as well as a CFIR interview guide tool designed for use in implementation studies [28]. The protocol was semi-structured, which allowed interviewees to guide the conversation to topics of importance to them, but also ensured relative consistency across interviews. Sample questions included: “What were some of the challenges or barriers that you faced in the implementation process? How did clinical processes and provider roles change over time?” All interviews were audio-recorded and transcribed verbatim.

Data were also drawn from observations of planning meetings. During or immediately following each meeting, observations were recorded as fieldnotes by the study PI. Fieldnotes described the subject matter of the meetings (gaps in clinical care, possible approaches to overcome gaps identified, etc.) as well as how and by whom information was communicated. Fieldnotes were typed and uploaded to the qualitative software management program, AtlasTI version 8.

To analyze study data, the study PI and a master’s level health services researcher first independently read through all interview transcripts and fieldnotes. They independently coded three observations and two interviews using a codebook developed for use in studies utilizing CFIR [31]. Specifically, coding sorted the qualitative data into CFIR constructs and domains. Additional codes were added as needed. Two study authors then met to compare coding and ensure that codes were understood and applied consistently. All study documents were then equally divided between two researchers for coding and review. Each transcript was coded by a primary coder, whose coding was carefully reviewed and queried by a secondary reviewer. The two researchers equally shared the roles of coder and reviewer in the overall data set. Inconsistencies in coding were resolved through consensus methods during the review process when the coder and reviewer met to identify and resolve discrepancies.

To analyze study data, coders identified frequently occurring, and particularly salient codes, and pulled all quotes associated with these codes. Quotes were then compared within codes, to identify key dimensions of the primary constructs identified, and across codes, to identify relationships between constructs. In the text, authors describe key events that made up the implementation process, and present illustrative quotes describing how and why events unfolded as they did.

Two authors independently mapped the processes and affiliated CFIR constructs to established ERIC implementation strategies [30, 31]. To translate local strategies into standard implementation science terminology, we: (a) describe the locally initiated strategy, (b) categorize the strategy within a CFIR framework and (c) match the local strategy to the ERIC implementation strategy with which it is most closely aligned [30]. Inconsistencies in coding were resolved through consensus. The Consolidated criteria for Reporting Qualitative research Checklist (COREQ) describes additional methodological detail (see Additional file 1)

## Results

**Fig. 1:**
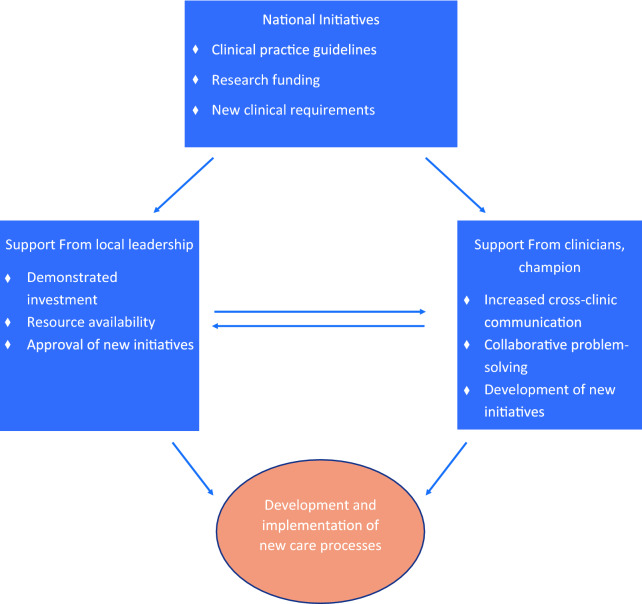
Pathways linking national to local initiatives to expand access to MOUD.

Drawing upon interviews with clinicians and administrators (n = 9) and ethnographic observation of planning meetings (n = 3) we describe the development of a Primary Care-Based Buprenorphine Clinic, which allowed patients to access buprenorphine for OUD outside of a specialty substance use disorder or mental health setting, and an E-Consult Service, which provides virtual consultation regarding OUD diagnosis and treatment to clinicians throughout the health care system, including in remote and rural locations. First, we detail how policy change at the national and local level altered clinicians’ perceptions of the need for expanded access to MOUD. We then describe how a model of care delivery was identified and staffing and resources secured. Finally, we describe how a self-appointed team increased staff investment in the change process by networking across clinical silos and bringing staff into the planning process. Figure 1 provides a visual display of the pathways linking national policy to local change processes. Table 1 maps the local actions and processes described in the text to CFIR constructs and ERIC implementation strategies.

**Table 1:** Processes mapped to established implementation constructs and strategies

What happened?	How would this action be classified by CFIR?	Which expert recommendation for initiating change (ERIC) implementation strategy best describes the action?
*National policy change *		
• Clinical practice guideline altered • VA national office published new clinical requirements	Outer setting (external policy and incentives)	Dissemination strategy • Mandate change
*Local actions and initiatives *		
• Champion participated in multiple, cross-clinic planning meetings • Champion researched intervention	Process (engaging the champion)	Implementation process strategy • *Champion activated*^a^
Within cross-clinic meetings, team: • Mapped current treatment capacity and care-gaps • Shared information • Strengthened communication networks • Promoted collaboration	Inner setting (networks and communication)	Implementation process strategies • Conduct a local needs assessment • Promote network weaving • Build a coalition
• Launched a process improvement team • Fielded a survey to identify supports needed for intervention	Process (planning)	Implementation process strategy • Assess for readiness and identify barriers and facilitators
• Identified new staffing source • Redesigned staff roles	Inner setting (available resources)	Integration strategies • Create new clinical teams • Revise professional roles
• Identified appropriate patient population • Identified a compatible care-delivery model	Inner setting (compatibility)	Integration strategies • Promote adaptability • Tailor strategies
• Leadership voiced support in a public forum	Inner setting (leadership engagement)	Capacity building strategy • *Showcase leadership support*^b^
• Hosted a summit to: ○ Brainstorm solutions ○ Solicit public commitment to participate	Process (engaging, learning climate)	Implementation process strategies • Build a coalition • Create a learning collaborative • Obtain formal commitment
• Launched an E-Consult service	Inner setting (networks and communication, available resource)	Integration strategies • Facilitation • Create new clinical teams • Tailor strategies • Obtain formal commitment • Promote network weaving

Implementation Processes Mapped to Consolidated Framework for Implementation Science (CFIR) Constructs and Domains and Expert Recommendation for Implementing Change (ERIC) implementation strategies. Proposed additions to the compilation of ERIC Implementation strategies are identified in italics and described below

^a^Champion Activated builds upon the sole existing implementation strategy referencing a site Champion (Champion Identified) to reflect (a) the Champion’s self-motivated assumption of the role and (b) how learning and collaboration catalyzed the clinician’s decision to assume the role

^b^Showcase Leadership Support identifies leadership voicing active support of an implementation initiative or process in a public forum as an implementation strategy that generated staff interest and commitment to participate in the new processes

### Setting the stage for practice change

In 2017, the VA and Department of Defense (DoD) jointly published clinical practice guidelines addressing the use of long-term opioid therapy for chronic pain [32]. Similar to 2016 Centers for Disease Control and Prevention guidelines on opioid prescribing, VA/DoD guidelines recommended routinely evaluating the need to continue long-term opioids for chronic pain with guidance to reduce or discontinue opioids when risks exceeded benefits [33]. While not addressing OUD treatment specifically, this guidance from the outer setting had significant implications for primary care clinicians’ perception of the need to offer MOUD in primary care. As one clinician noted, implementing the new guidelines necessarily meant that, “…we’re going to unearth more people with opiate use disorder and [there would]…be a need to offer them options for a treatment.” At the time, no providers in this health system were prescribing buprenorphine within primary care, although several had completed the DEA waiver training. While patients could access specialty care according to the VA’s Stepped Care Model for Opioid Use Disorder [4], establishing care with specialty OUD services required patients to self-schedule a lengthy intake visit limited to certain time slots during the week. This requirement presented barriers for some patients. Moreover, primary care providers lacked a venue to communicate or consult with specialists in OUD treatment outside of the electronic health record (EHR). These gaps meant that, despite pressures to taper long-term opioids, clinicians had limited options to treat patients who began to exhibit symptoms of OUD during the taper process. As one clinician remarked, “we were consistently running into this situation of these patients that we were kind of on the treadmill with and getting nowhere.”

A second outer setting change, initiated by local primary care leadership and implemented by the Clinic Practice Manager, further heightened clinicians’ awareness of the lack of OUD treatment capacity within primary care. The new initiative linked opioid metrics (e.g., percent of completed urine drug screens, reviews of the state prescription drug monitoring program database, and naloxone prescriptions) with physicians’ performance pay. In response, one primary care clinician described a group email thread he had sent to local leadership asking what would be done to address a patient with OUD identified through such processes, “I…raised the question… ‘should we really…[be] putting effort into identifying more people if we don’t put parallel effort into making more treatment accessible to them?… I kind of felt that it wasn’t ethical to… identify people without having treatment options available.” The change in national guidance and local performance pay incentives regarding opioid prescribing highlighted gaps in MOUD treatment availabilty and helped to alter clinicians’ perceptions of the need for a change.

### Starting a primary care-based Buprenorphine Clinic

Belief in the need for change was a necessary but not sufficient pre-condition for practice change to take place. What was crucial in this context, as has been identified in others, was the willingness of a Champion to take on the heavy lifting of moving the clinic from idea to reality [19, 34, 35]. At first, no one stepped forward to lead change efforts despite a general consensus that increased access to MOUD was needed and that the best next step would be to increase MOUD availability in the primary care setting. As one clinician described, “I think if everybody was lukewarm and there wasn’t somebody that was [really pushing]… I mean, everybody’s busy, there’s a billion other things to do. If you have many lukewarm folks about a particular topic, there’s no way for people to overcome the activation energy it’d take to…do something new.” Another clinician, who eventually took on the role of Champion, concurred, while also acknowledging that committing to the role had not happened immediately, “It was one of those things where it’s like a good idea, but no one really has… the time or capacity to really take it on. And, I put myself in that category also, for a while…” Yet as time went on, she became increasingly enthusiastic, in part due to fortuitous participation in a federally-funded systematic review addressing barriers to OUD treatment [20]. As she described, “[of] everything I’m doing, I now feel the most passionate about this one thing… From a public health and…social justice standpoint… it’s very clear to me that this is…a service [primary care buprenorphine] that we should be providing.” Her research coupled with the work of planning and soliciting support for expanded access to MOUD activated this clinician to take on the role of Champion.

Beginning in 2017 and through 2018, the Champion began meeting with specialists and administrators to understand where and how MOUD was already being offered within the larger health care system and what steps would be needed to start prescribing buprenorphine within the medical center’s primary care clinic. These meetings helped generate momentum for the idea of creating a Primary Care-Based Buprenorphine Clinic and obtain buy-in from stakeholders in the substance use disorder, pain management and mental health clinics, the other locations offering OUD treatment with buprenorphine. Seeing that some momentum was building encouraged other primary clinicians who were interested, but did not have time to do this groundwork themselves, to lend their support for change. The Champion, a small number of other interested primary care clinicians, the Clinic Practice Manager, a specialist from the substance use disorder clinic, and a pharmacist began meeting monthly to discuss how to overcome logistical barriers to implementing a new Clinic. Barriers discussed in these meetings included limited clinic space, staffing issues, and determining the future clinic’s patient capacity and scope, “There was a lot of, like…we want to do this, but who’s going to do it and how’s it going to be done?” The Champion was hesitant to lead the clinic on her own, “[It]…felt a little daunting just to say, like I’m going to do it all!” A turning point came when the pharmacist suggested that a second year Pharmacy Resident rotate in the clinic to gain OUD treatment experience (under the supervision of a Pharmacy Preceptor) and a second primary care clinician committed to co-directing the clinic with the Champion (hereafter referred to as the Clinic Co-Director). With this staffing in place, the team finalized the model to be implemented for buprenorphine care delivery: a clinician-pharmacy collaborative care model.

This model had precedent in the VA system, in which clinical pharmacists and primary care providers often collaborate on treatment plans for patients with chronic diseases such as diabetes, high blood pressure or chronic pain [36, 37]. In order to preserve the clinic’s capacity for new patients, the Champion or Clinic Co-Director would meet with patients initially to outline the care plan, and follow-up visits would be conducted by the Pharmacy Resident (with oversight from Champion or Clinic Co-Director and the Pharmacy Preceptor). Although the Pharmacy Resident could not prescribe buprenorphine, it was in the resident’s scope to ask patients about medication effectiveness, adherence, and side effects as well as order urine drug screens for treatment monitoring. The model was consistent with the primary care clinic’s established function as a teaching clinic and provided learners with substance use disorder treatment experience, “this gives the residents a leg up…when they graduate…they already have that little bit of experience with the SUD clinic that could be transcribable into the full practice.” Although the learners in this case would be pharmacy residents (rather than medical residents), the fact that the proposed clinic would meet an educational need helped garner support for the model from local primary care leadership. Concretely, support from the Clinic Practice Manager consisted of dedicating physical space for the clinic one morning/week and allowing for a reduction of about 100 patients in their usual primary care panel for both the Champion and Clinic Co-Director (without additional compensation or protected time). Nursing staff also agreed to support the clinic with patient check-ins and rooming, although the Champion and Co-Director pledged to provide some of this support themselves to avoid over-burdening nursing staff.

Initially the clinic planned to exclusively treat patients who were in sustained remission from OUD transferring from specialty care. OUD specialists viewed this as a beneficial expansion of “step-down” services and an appropriate starting point for primary care clinicians who lacked buprenorphine prescribing experience. As one pharmacy resident described, “…for us starting out… you want patients who have proven to be very stable and not had issues…” Such patients were thought to require fewer supportive services and less frequent touch-points, a treatment approach that was seen as more compatible with primary care practice, which lacked the ability to provide intensive case management. Further, it was decided that patients’ OUD care would be maintained by clinicians in the buprenorphine clinic, rather than transferred back to patients’ primary care clinician. In the clinic planning process, primary care clinicians and specialists in OUD agreed to maintain open lines of communication and discuss patient transfers back to a higher level of care—specialty substance use disorder treatment—when appropriate. In January of 2019 the Primary Care-Based Buprenorphine clinic was launched.

### Expanding capacity and scope

About a year after the Primary Care-Based Buprenorphine Clinic had been initiated, the core team comprised of the Champion, Clinic Co-Director, other interested clinicians and pharmacists turned to the question of how to further expand access to OUD treatment for patients seen elsewhere in the health care system (e.g., for hospitalized patients, those seen in rural clinical settings). Such settings lacked a consistent process for providing MOUD and engaging patients in ongoing treatment, and some clinics lacked buprenorphine prescribing capacity. One clinician proposed, and received support from leadership to host, a half-day “Buprenorphine Summit” in January of 2020, which was intended to engage clinicians throughout the system in discussion of gaps in treatment availability, and strategies to overcome them. Fortuitously, just before the Summit was scheduled to convene, national VA leadership disseminated a directive mandating that all VA sites begin prescribing buprenorphine within 60 days of receipt of the notice [38]. While in the past local leadership had been hesitant to integrate buprenorphine treatment into clinical settings beyond specialty SUD, mental health, and the nascent Primary Care-Based Buprenorphine Clinic, “leadership was concerned that if we open up care for buprenorphine that…will gobble up appointments and we won’t be able to provide care for other veterans who want to get into the VA…,” with the publication of the notice, “there was impetus to move forward.” The national directive also had implications for local funding decisions, as one clinician described, “If this is a priority for the VA, you can justify the resources for that.” Support from local leadership was evident at the Summit. Not only were leaders from primary care, mental health, emergency medicine, pain management, and pharmacy in attendance, but as the Summit convened, the health system chief of staff provided introductory remarks emphasizing her strong support for the Summit’s goals.

A key component of the Summit was discussion of a series of hypothetical cases, designed to highlight existing gaps in MOUD availability, and the barriers to connecting patients to treatment. For instance, one case presented a patient with OUD receiving care in a rural clinical setting who lacked a clinician certified to prescribe buprenorphine. Another case discussed how to continue buprenorphine for a patient who started buprenorphine while hospitalized but lacked an outpatient provider. The case discussion provided a forum for clinicians to consider the appropriate clinical home for patients with diverse needs and come to agreement on responsibilities across primary care and specialty clinics, which had been somewhat poorly defined in the past. The public nature of the forum also placed a subtle group pressure on clinicians to step up to fill these gaps as they were able; at one point the Champion asked whether anyone would be willing to volunteer to fill a particular role and waited through silence until someone came forward.

Finally, participants reviewed and discussed a variety of possible approaches to expanding access to buprenorphine to satellite primary care clinics and other clinical settings. Prior to the Summit, the organizers had distributed an informal survey via email to clinicians to inquire about their interest in and current use of buprenorphine for OUD. The email also asked what kinds of supports would increase clinicians’ willingness to prescribe buprenorphine. During the Summit, results of the survey were used as a starting point for the discussion. The Champion presented several possibilities (e.g., an E-Consult service, a hub and spoke model, visiting experts to assist outlying or rural clinics), and other attendees generated additional ideas (e.g., a buprenorphine road show, a mock drug enforcement agency audit). The E-Consult generated the most enthusiasm from attendees and the decision was made to move forward with planning. By providing space for participants to brainstorm solutions, the Summit encouraged collaborative problem solving and spurred investment in the proposed initiatives by bringing participants into the planning and development process.

### The E-Consult service

In follow-up planning meetings, the core team discussed E-Consult staffing, scope, design and implementation. Fundamentally, it was decided that the E-Consult would be created within the EHR, made available to all clinicians within the health system and encourage questions regarding how to establish an OUD diagnosis, initiate buprenorphine and approach care transitions, thereby providing, “immediate access mentoring.” The Champion and Clinic Co-Director took ownership of the E-Consult process including the responsibility to respond to consults within the EHR. Because the Primary Care-Based Buprenorphine Clinic was not yet at capacity, the Champion and Clinic Co-Director did not receive additional protected time to manage the E-Consults and instead wrapped this work into their other clinical responsibilities. As a compliment to the E-Consult process, the core group established a twice weekly 30-minute meeting to address questions such as, “what’s the appropriate avenue for this patient, I’m considering buprenorphine therapy,” or, “they’re on buprenorphine therapy and I’m not sure what to do with them.” Attended on a voluntary basis by other clinicians from primary care, mental health, the substance use disorder clinic, and the pain management clinic, this ad hoc interdisciplinary team worked together to determine the appropriate clinical care setting for each patient:

“There’s fluidity in that, so if it doesn’t seem to work in one setting maybe we can move into another…we have…buy in from [the specialty substance use disorder treatment program], from primary care, from inpatient service, we’re trying to get ED involved… the pain clinic… the patient could fit into any of those situations and…we have a mechanism to talk it over and try and make a fit for the patient.”

The E-Consult Service facilitated care coordination by creating a centralized hub for case discussions, thereby addressing multiple systemic issues from lack of communication among clinicians to a lack of treatment capacity in some clinical settings. As one clinician described, “we’ve kind of broken the barriers and silos around care for these patients.” While the resolution for some E-consults might be to schedule a visit in the Primary Care-Based Buprenorphine Clinic, the E-consult process also facilitated patient hand-offs to the substance use disorder and pain management clinics. In some cases, the group might also provide recommendations for ongoing treatment in primary care.

### Taking stock

The Primary Care-Based Buprenorphine Clinic saw its first patients in early 2019 and the E-Consult process was finalized just over 1 year later, in February of 2020. Since that time, the landscape of MOUD treatment availability across the health care system has transformed. The Primary Care-Based Buprenorphine Clinic has become well-established, continuing to operate one morning/week, with plans to expand to another half-day. With increased experience, the clinic no longer only serves as a step-down clinic for “stable” patients with OUD, but rather functions as a bridge clinic for patients seen in the hospital or emergency department who need a higher level of care (e.g., in the substance use disorder clinic) but are awaiting appointment availability. In the 3-years the clinic has existed, it has served 65 unique patients and provided training opportunities for seven clinical pharmacy residents, 24 psychiatry residents, and other learners including addiction medicine and palliative care fellows. While Internal Medicine residents do not rotate with the clinic on regular basis, they often refer patients to the clinic and attend the twice-weekly team meetings. This form of collaboration has provided additional opportunities for teaching. The E-Consult Service continues to meet twice weekly and has consulted on 225 patient cases, helping to facilitate initiation or continuation of buprenorphine treatment and linking patients to the most appropriate sites of care. Moreover, with a shift to telework following the onset of COVID-19, the twice weekly case discussions shifted from in-person to virtual meetings, which allows participation from staff at remote clinic sites, thereby furthering the overall educational impact. Over the 3-year period in which these new clinical processes were developed and implemented, the health system has increased the percentage of patients with OUD on medication from approximately 35% to more than 50%. While this improvement cannot be attributed to the Primary Care-Based Buprenorphine Clinic and E-Consult alone, these services are now an integral part of the health system’s efforts to expand MOUD access.

For one member of the team, the intentionally slow, collaborative approach to practice change had been essential to the ultimate success of these efforts:

I think [the Champion’s] appropriately, taught me something about…building the community of providers that you need… hearing all the different sides of this and making sure everybody’s on board and taking…a patient approach... That’s the way to…have a longer standing…program in place that’ll fit in well with the rest of the organization.

While this particular clinician had initially advocated for a more “rabble-rousing” approach to system change, he later reflected that such an approach may not have yielded the institutional buy-in and program sustainability of the more cautious, methodical approach ultimately taken.

## Discussion

This study revealed the processes through which a self-appointed local team developed, established broad support for, and successfully implemented new processes of care that expand access to an essential medical treatment. First, changes in national guidelines and policy led to increased support from local leadership, which resulted in greater resource availability and the approval of new initiatives. For the Champion and other clinicians, changes to national guidelines and local policy increased perception of the need for practice change as well as desire to participate in the planning process. A self-appointed team, activated by local incentives and an awareness of barriers to MOUD access, engaged in cross-clinic meetings and information gathering to identify a model of care delivery that would fill gaps in treatment availability, was compatible with existing clinical practice and supported by leadership. Finally, the team increased staff investment in these change efforts by bringing them into the planning process and encouraging collaborative problem solving.

This study contributes to implementation science in several ways. First, a common critique of implementation strategies is that the change process is often externally initiated (pushed) rather than internally motivated (pulled) [39]. By detailing the strategies underlying a locally-initiated change process, this study reveals how systems lacking the resources of an external implementation team may seek to create change from within. While many of the strategies we describe are consistent with, and could be successfully utilized by, implementation teams partnering with internal facilitators, we suggest that the processes we describe differ in the source of the motivation for change; internal facilitation is most often motivated by an external partnership, whereas the processes we describe emerged at the local-level. Future work may consider the extent to which there may be differences in the sustainability of implementation efforts that are internally-driven relative to those initiated by external sources [40]. Second, our results revealed the possibility of two previously unidentified implementation strategies: Champion Activated and Showcase Leadership Support (Table 1). The first new strategy references that, in our site, the Champion was not appointed to the role, but rather became self-motivated to take it on following a process of activation. This contrasts with the ERIC strategy, “Identify and Prepare Champion,” which assumes a Champion is selected or assigned to the role. Future research might consider what constellation of factors could explain this process of activation and self-appointment, and how to lay the groundwork for such activation to take place. Second, we suggest that visible support from leadership may be another previously unidentified facilitator, and one that could be easily replicated in other settings. In our site, this took the form of leadership presence and verbal endorsement at a large planning meeting, and support for protected time for the Champion and Clinic Co-Director. Future research could examine other possible strategies for showcasing leadership support, as well as how support from leaders at other levels of leadership may differentially influence staff motivation and buy-in.

By providing a narrative account of the change-process from the perspective of those who initiated it, this study goes beyond identifying which strategies affect change, to instead build understanding of how and why implementation strategies work [41, 42]. Future research utilizing qualitative comparative methodology should consider the extent to which the CFIR constructs and implementation strategies identified here are also present in locally-initiated implementation efforts in other settings, and which are necessary for implementation success.

This research also has implications for expanding accessibility to MOUD. One commonly advocated approach is to offer buprenorphine in primary care settings, rather than limiting buprenorphine prescribing to specialty substance use disorder treatment settings [43]. In our site, a collaboration with pharmacy with an emphasis on the trainee experience enabled primary care to institute a dedicated clinic offering buprenorphine to patients. This model of care delivery was consistent with the teaching mission of the institution, drew upon a pool of motivated clinicians and won the support of leadership. Other academic health care systems seeking to expand access to MOUD in primary care may consider this approach, which has the benefit of ensuring that learners acquire the skills and confidence to incorporate MOUD prescribing into their practice following training. Another lesson relates to our site’s Champion. While prior research has identified the importance of a clinical Champion to drive adoption of MOUD [19, 35], little is known about how a Champion is activated to assume the role. Our Champion was primed through national and local policy change that increased tension for change, bolstered through well-timed participation in a federally-funded research project addressing barriers to MOUD and solidified through months of planning and collaboration with other stakeholders. Other systems could work to set the stage for the emergence of a Champion by enacting supportive policies, leveraging related research activities, and allowing clinicians the time and resources needed to meet and strategize about how best to enact changes.

This study is not without limitations. First, the study took place in a single academic medical center within a fully integrated healthcare system; it is unknown how the change-processes we document would apply to other settings. Second, the study was conducted during a time when there was strong support for expanding access to MOUD from leaders within the healthcare system as well as from the popular press. In the absence of such a supportive climate, outcomes of the processes described may have yielded different results. Finally, absent a comparison site, we cannot be certain that the increased access to MOUD we observed is attributable to these new care processes; it is possible that access to MOUD would have increased across this time period in the absence of these efforts. Future research is needed to compare outcomes across sites to determine the significance of the processes and strategies deployed in this setting.

## Conclusions

As the opioid crisis continues, expanding access to MOUD remains a key priority for health care systems. Building upon implementation research that has described externally-facilitated strategies to expand access to MOUD, this study reveals how a self-appointed local team developed and built widespread support for new approaches to care delivery that were tailored to local needs and well-positioned for sustainability over time.

## Supplementary Information

**Additional file 1.** COREQ (consolidated criteria for reportingqualitative research) checklist.

## Data Availability

Study data are available from the corresponding author on reasonable request.

## Abbreviations

MOUD: Medication treatment for opioid use disorder
OUD: Opioid use disorder
SUD: Substance use disorder
CFIR: Consolidated Framework for Implementation Research
ERIC: Expert Recommendations for Implementing Change
DoD: Department of Defense
